# Racial and Ethnic Differences in the Effects of Prescription Drug Monitoring Program Laws on Overdose Deaths in the United States

**DOI:** 10.1111/1468-0009.70057

**Published:** 2025-10-13

**Authors:** SPRUHA JOSHI, VICTORIA A. JENT, SNEHA M. SUNDER, KATHERINE WHEELER‐MARTIN, MAGDALENA CERDÁ

**Affiliations:** ^1^ University of Michigan; ^2^ NYU Grossman School of Medicine

**Keywords:** overdose, opioids, prescription drug monitoring program

## Abstract

**Policy Points**

- State “must‐query” prescription drug monitoring programs (PDMPs) were associated with increased overdose deaths, suggesting these policies may have unintended consequences.
- Black and Hispanic populations experienced disproportionately higher increases in overdose deaths following must‐query PDMP adoption, highlighting that these policies may contribute to health disparities.
- Addressing systemic inequities in health care access and substance use treatment may help supplement the effective components of PDMPs, ensuring that these policies reduce rather than exacerbate overdose deaths.

**Context:**

Despite recent declines in national overdose deaths, these reductions have not been equitably experienced. Black and Hispanic communities continue to face rising rates of opioid‐related mortality, even as overdose death rates among White individuals have begun to decline. One of the most widely implemented policy responses to the overdose crisis has been the adoption of prescription drug monitoring programs (PDMPs), particularly “must‐query” mandates requiring prescribers to consult the PDMP before issuing controlled substances. However, limited research has examined whether the impact of these mandates varies by race and ethnicity.

**Methods:**

We used restricted‐use National Vital Statistics System data from 2013 to 2020 to estimate county‐level overdose mortality stratified by drug type and race and ethnicity. We categorized deaths as follows: (1) all drug overdoses, (2) all opioid overdoses, and (3) natural/semisynthetic opioid overdoses. Exposure to must‐query mandates was modeled as the proportion of the prior year during which mandates were in effect. Using Bayesian spatiotemporal models with county random effects and spatial autocorrelation, we estimated relative rates (RRs) for each outcome overall and by race and ethnicity, adjusting for state policies and sociodemographic characteristics.

**Findings:**

Must‐query mandates were associated with increases in overdose deaths across all groups, with the largest relative increases among Hispanic (RR = 1.32, 95% credible interval [CrI]: 1.21‐1.44) and Black individuals (RR = 1.23, 95% CrI: 1.14‐1.33) compared with White individuals (RR = 1.14, 95% CrI: 1.10‐1.19). These increases were also observed among Black and Hispanic individuals for natural/semisynthetic opioid overdoses.

**Conclusions:**

PDMP must‐query mandates are not uniformly protective across racial and ethnic groups. Increases in overdose mortality following adoption, particularly among Black and Hispanic populations, underscore the need to evaluate drug policies through an equity lens and consider broader structural determinants of health that shape their effectiveness.

The united states is in the midst of a drug overdose epidemic. the drug overdose death rate in 2023 was 4.3‐times higher than in 1999,^1^ and more than 75% of drug overdose deaths in 2023 involved an opioid.^2^ The drug overdose crisis in the United States has evolved through distinct waves since the late 1990s. Initially driven by a dramatic rise in the prescribing of opioid painkillers, most notably oxycodone and hydrocodone, the epidemic was fueled by aggressive pharmaceutical marketing and the medical community's shifting attitudes toward pain management. As regulations on prescription opioids tightened and, in turn, the availability became restricted in the early 2010s, many individuals transitioned to heroin and subsequently to illicitly manufactured fentanyl.

Historically, overdose mortality was highest among White populations, particularly in rural and suburban areas. However, recent years have witnessed a distinct shift in groups bearing the greatest burden. Black and American Indian/Alaska Native communities now experience some of the highest overdose death rates, reflecting broader patterns of structural disadvantage and differential access to prevention and treatment resources. Although preliminary 2024 data show a national decline in overdose deaths, this progress has been unevenly distributed. The decline has primarily benefited White populations, whereas overdose mortality among racial and ethnic minority groups has remained stagnant or continued to rise. As a result, racial and ethnic disparities in drug overdose deaths have not only persisted but widened, underscoring the urgent need for targeted, equity‐focused public health interventions.

Given these shifting trends, in 2023, the overdose death rate of Black individuals was 33% higher than that of White individuals, whereas American Indian/Alaskan Native individuals had the highest drug overdose death rate of any group: 83% higher than White individuals.^1^ A majority of opioid overdoses in 2023 involved non‐methadone synthetic opioids such as fentanyl,^1^ and the remaining deaths were caused by prescription opioids (18%), and the remaining 7% involved heroin.^2^ Although a majority of deaths were caused by nonprescription opioids, disaggregating these deaths by race and ethnicity reveal concerning patterns. In 2013, 82.2% of all opioid overdoses were among White individuals, 7.7% were among Black individuals, and 7.8% were among Hispanic individuals. By 2020, the proportion of total opioid overdoses that were among White individuals had decreased to 68.8%, whereas the proportions that were among Black and Hispanic individuals increased to 16.9% and 11.6%, respectively, uncovering increases among vulnerable groups.

In response to the opioid overdose crisis, states have adopted a range of policy and legislative developments. One of the most widespread policy responses during the first wave of the overdose crisis involved the adoption of state prescription drug monitoring programs (PDMPs). PDMPs are a supply‐side form of opioid regulation collecting prescribing information on controlled substances such as prescription opioids. The first drug monitoring program was implemented in 1918 in New York to regulate prescriptions for opium, cocaine, codeine, heroin, and morphine and was terminated in 1921. PDMPs have evolved over the last century, and, by 2021, all 50 states and the District of Columbia had an operational PDMP.^3^ Although PDMPs in the United States have commonalities in terms of centralized statewide data systems that electronically transmit prescription data, the administrative features of PDMPs, including regulatory agency, type of data collected, who has access, and mandated requirements to check the PDMP before prescribing and dispensing, have varied substantially among states and over time. Dispensers can use PDMPs to ensure that prescriptions align with clinical guidelines and detect suspicious behavior, such as doctor shopping. Law enforcement agencies may use PDMP data to uncover fraudulent prescription activities or the diversion of prescription drugs.^4^

Research on the effects of PDMPs on fatal overdoses has yielded mixed results. Some studies have found a decrease in overdose deaths related to prescription opioids after PDMPs were implemented.^5^, ^6^, ^7^ In contrast, another study reported a significant rise in total drug overdose rates,^8^ and other studies have found no meaningful link between PDMPs and prescription opioid‐related overdose deaths.^9^, ^10^ Some evidence suggests that “must‐query” PDMPs, which mandate that prescribers check the database before prescribing certain controlled substances, are most effective in lowering prescribing rates.^11^, ^12^, ^13^ However, research examining the impact of “must‐query” PDMPs on overdose have also yielded inconsistent findings. Some evidence suggests that must‐query PDMPs contributed to a reduction in prescription opioid deaths,^11^, ^14^, ^15^ as well as increased rates of overdose death related to heroin and cocaine.^14^ Other studies have reported increases in opioid‐related overdoses following adoption of must‐query PDMPs,^16^, ^17^, ^18^, ^19^ and some found no change in the number of opioid‐related deaths.^20^ Another important source of discrepancy in findings across studies examining the association between PDMP implementation and overdose rates is the timing of the studies. Earlier studies, conducted when prescription opioids were the primary driver of overdose deaths, may yield different conclusions than those conducted in later years marked by the rise of heroin and fentanyl. This temporal shift in the specific opioids involved, as well as in the populations most affected, hinders the inability to make direct comparisons.

Most studies to date focus on overall population rates with no consideration for heterogeneity across racial and ethnic groups. One study found that PDMPs with must‐query requirements were associated with a greater reduction in opioid dispensing in Black patients than in White patients, even though Black patients nationally already had lower rates of nonmedical prescription opioid use and overdose.^21^ Absent from the literature is the examination of the impact of PDMPs on fatal overdose rates by race and ethnicity. Racial and ethnic disparities in opioid overdose mortality are rising: nationally, drug overdose death rates recently increased among Black, Hispanic, and Indigenous populations even as rates among White individuals declined, and Black communities experienced a 49% jump during the COVID‐19 surge alone.^22^ In particular, natural and semisynthetic opioid mortality has continued climbing among Black American individuals at an average of 17% per year from 2013 to 2020, whereas rates among White individuals plateaued. Given that the rates of fatal overdose caused by natural semisynthetic opioids are rising among non‐White populations, it is critical to understand the impact that PDMPs have among these populations. Without this evidence, we risk perpetuating policies that fail to address, or even exacerbate, racial disparities in opioid‐related harm. It is important to understand the influence of PDMPs across racial and ethnic groups not only because of observed differences in opioid use patterns but also because of long‐standing inequities in health care access, pain management, and substance use treatment. Policies like PDMPs are not implemented in a vacuum; they intersect with existing structures and biases across multiple levels such as other policies, clinical recommendations, and long‐standing structural racism. Despite the widespread adoption of PDMPs, little is known regarding whether these policies, particularly must‐query provisions, mitigate or exacerbate such disparities. Without evidence on their potential differential impacts, there is a risk that PDMPs may unintentionally reinforce or widen existing inequities in overdose mortality. Understanding whether PDMPs are an effective tool for reducing overdose mortality equitably, or whether additional or alternative interventions are needed to address racial and ethnic disparities, can influence policy decisions regarding the modification or replacement of must‐query PDMP provisions in the context of evolving overdose trends.

We build on previous literature by filling these gaps. First, we look at the impact of PDMP must‐query requirements on overdose deaths nationally from 2013 to 2020, characterizing overdose deaths in the following three ways: (1) total overdose deaths, (2) total opioid overdose deaths, and (3) natural and semisynthetic opioid overdose. Second, we examined the association between PDMP must‐query requirements and overdose outcomes by race and ethnicity, examining the relationship across the following groups: overall, non‐Hispanic White, non‐Hispanic Black, and Hispanic. Our study addressed the following questions: (1) What is the relationship between state‐level PDMP “must‐query” mandates and county‐level fatal overdose counts by (a) any drug overdose, (b) any opioid overdose, (c) natural and semisynthetic opioid overdose other than heroin?; and (2) How does this relationship vary by the race and ethnicity categories of (a) White, non‐Hispanic; (b) Black, non‐Hispanic; and (c) Hispanic?

## Methods

### Outcome Measures

We used the National Vital Statistics System's restricted‐use detailed multiple cause‐of‐death (MCOD) mortality files to tabulate county‐level drug‐related overdose deaths from 2013 to 2020.^23^ Codes from the *International Classification of Diseases, Tenth Revision* were used to identify drug overdose deaths (X40‐44, X60‐64, X85, and Y10‐14 as underlying causes). MCOD codes were used to define opioid overdose deaths (T40.0‐T40.4 and T40.6) and natural semisynthetic opioid overdose deaths (T40.2), based on national poisoning surveillance standards for opioid overdose.^24^ Deaths were stratified by race and ethnicity, and annual counts were aggregated to the county and year of death. Owing to boundary changes during the study period, 13 counties in Alaska were condensed to be one geographic unit.

Using data from American Community Survey (ACS) 5‐year estimates, counties included within the analyses were restricted to counties having a minimum of 1,000‐person population of Black and 1,000‐person population of Hispanic individuals throughout the study frame (*n* = 1,340).

### Exposure

We accounted for the operationalization status of electronic PDMPs with a state law that require providers to check PDMPs before prescribing opioids to patients, also known as “must‐query” laws.^25^ We obtained effective dates (i.e., dates that PDMP query mandates began) of state laws requiring prescribers to check PDMPs before prescribing opioids (see Horwitz and colleagues^25^ for methods). “Must‐query” laws were defined as any law that required any prescriber to check the PDMP before prescribing any category of controlled substances likely to contain prescription opioids. The definition included laws with narrow and common exemptions, such as palliative prescriptions, but excluded laws applying only to limited settings such as pain clinics. In almost all cases, must‐query mandates were enacted after the respective PDMPs were considered fully operational and electronically accessible by all users. Our measure of exposure was the proportion of the year that these laws were in effect, with a 1‐year lag.

### Covariates

We controlled for other relevant state laws by coding these covariates as the proportion of the calendar year that each law was in effect in that state, capturing both presence and timing of enactment. This includes overdose Good Samaritan laws,^26^ naloxone access laws, Medicaid expansion, and pain management clinic (PMC) laws with and without criminal penalties for violations.^27^ Policy variables, including the PDMP measure, were lagged by 1 year. Covariate policy data were obtained from the Prescription Drug Abuse Police System (PDAPS)^28^ and Kaiser Family Foundation (Medicaid expansion).^29^

County‐level ACS 5‐year estimates were used within the models: age (percentage of the populated aged 0‐17, 18‐24, 25‐44, 45‐64, and >65 years), gender composition (percentage of the population who identified as male), racial and ethnic composition (percentage of the population who identified as non‐Hispanic White, non‐Hispanic Black, Hispanic, American Indian/Alaska Native, or “other,” defined as Asian, Pacific Islander/Native Hawaiian, and other reported race), socioeconomic conditions (percentage of families living in poverty within the last 12 months and percentage unemployed in civic labor force, median household income), and population density (1,000 persons per square mile).^30^

Additionally, we included the overall county‐level mortality rate per 1,000 residents using the total number of deaths per county‐year recorded in National Vital Statistics System's restricted‐use MCOD files.

### Spatiotemporal Models

We estimated county‐by‐year RRs for the following: (1) any drug overdose, (2) any opioid overdose, and (3) natural and semisynthetic opioid overdose other than heroin. Each outcome was analyzed for the following populations: (1) total; (2) White, non‐Hispanic; (3) Black, non‐Hispanic; and (4) Hispanic.

We used a Bayesian spatiotemporal model (an expanded Besag‐York‐Mollié model) specification that included state fixed‐effects and county‐level random intercepts.^31^, ^32^ The model also includes random slope time trend for each county. The model incorporates a conditional autoregressive spatial random effect to account for lack of independence of race‐stratified overdose counts in spatially contiguous counties and to avoid biases caused by small area effect.^33^, ^34^ Using both spatial and nonspatial error terms, this model accounts for overdispersion. All analyses were performed using R (R Foundation for Statistical Computing) with the R‐INLA (R‐INLA Project) package.^35^, ^36^ The model estimated posterior means and 95% credible intervals (CrIs) for the PDMP predictor variable and covariates.^37^ Relative rates (RRs) are reported by exponentiating estimates. These estimates are comparable with the estimates from difference‐in‐difference approaches while using the advantages of spatiotemporal modeling.

### Sensitivity Analyses

We replicated the aforementioned analyses for all stratified groups and outcomes with a different definition of counties to be included in analyses. In the sensitivity analyses, we included 1,000 persons for the entire study period, using data from ACS 5‐year estimates at the county level (n = 3,095). For race‐stratified analyses, counties were restricted to a minimum of 1,000 persons of a specific race or ethnicity. The stratified analysis for White individuals includes 2,809 counties, for Black individuals includes 1,396 counties, and for Hispanic individuals includes 1,492 counties. Owing to unequal national distribution of the Black population within the United States, some states were removed from the analyses. We removed Wyoming, Idaho, and South Dakota because of the low population of Black residents at the state level.

## Results

### Descriptive

During the study period, that is, 2013 to 2020, overdoses deaths increased twofold. Although a majority of opioid overdoses in 2020 involved non‐methadone synthetic opioids,^1^ the remaining deaths were caused by prescription opioids (18%), and the remaining 7% involved heroin (Figure 1).^2^ Whereas a majority of deaths were caused by nonprescription opioids, disaggregating these deaths by race and ethnicity reveal concerning patterns. In 2013, 82.2% of all opioid overdoses were among White individuals, 7.7% were among Black individuals, and 7.8% were among Hispanic individuals. By 2020, the proportion of total opioid overdoses that were among White individuals had decreased to 68.8%, whereas the proportions that were among Black and Hispanic individuals increased to 16.9% and 11.6%, respectively, uncovering increases among vulnerable groups (Figure 2).

**Figure 1:**
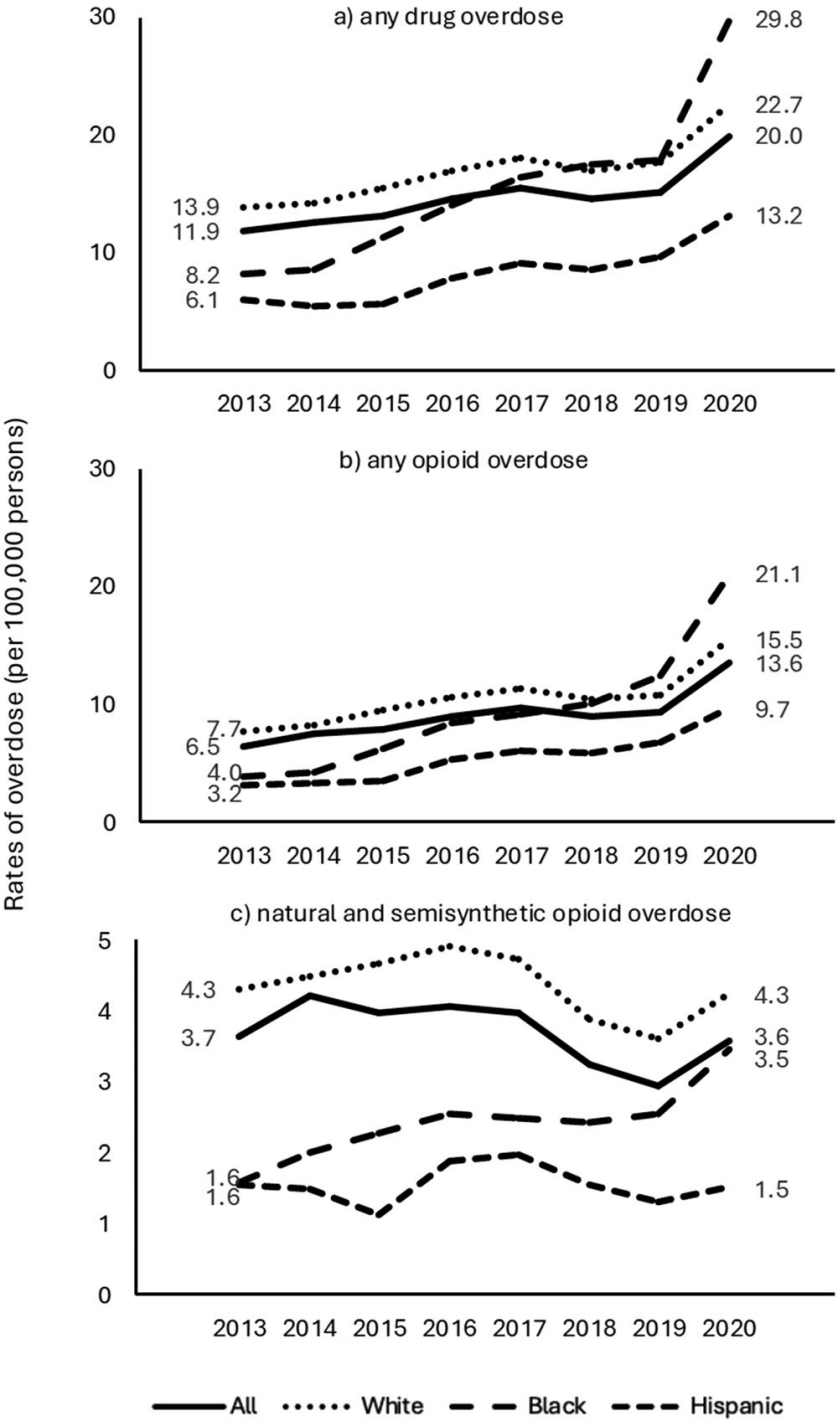
Annual Rates of Overdose by Drug Type and Race and Ethnicity, 2013 to 2020

**Figure 2:**
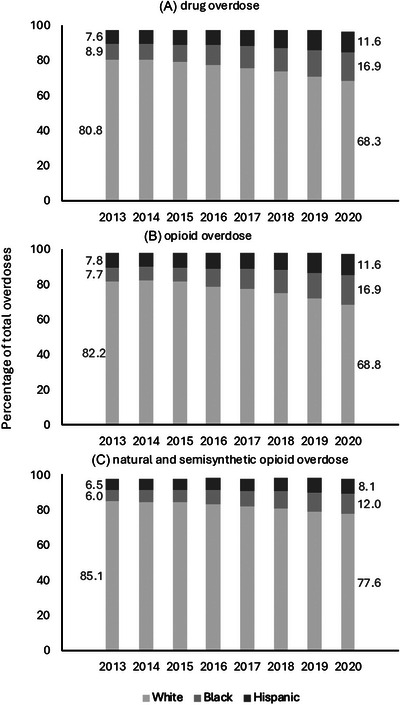
Annual Percentages of Overdose by Drug Type and Race and Ethnicity, 2013 to 2020

Table 1 compares the study county characteristics in 2013 and 2020. In 2013 to 2020, the number of states with PDMP must‐query laws grew from two to thirty‐six (Table 1). Table 2 compares the study county characteristics for each of the race‐specific outcome samples (Table 2).

**Table 1:** County Characteristics (Median and IQ Range), United States, 2013 to 2020

Characteristic	Total Counties (*n* = 3,134)	Study Counties^a^ (*n* = 1,340)
	Median (IQ Range)	Median (IQ Range)
Age group %, y		
0‐17	22.5 (20.5, 24.4)	22.8 (20.9, 24.6)
18‐24	8.1 (7.2, 9.4)	8.8 (8.0, 10.1)
25‐44	23.3 (21.4, 25.2)	24.7 (23.2, 26.5)
45‐64	27.5 (25.8, 29.1)	26.8 (25.1, 28.2)
65 and older	17.4 (14.8, 20.2)	15.5 (13.3, 17.8)
Sex %		
Female	50.4 (49.5, 51.1)	50.8 (50.0, 51.4)
Male	49.6 (48.9, 50.5)	49.2 (48.6, 50.0)
Race and ethnicity %		
White, non‐Hispanic	84.3 (65.3, 93.0)	74.4 (59.2, 85.4)
Black, non‐Hispanic	2.1 (0.6, 10.0)	7.6 (3.0, 18.5)
Hispanic	3.9 (2.0, 9.3)	5.9 (3.4, 12.4)
Native American	0.3 (0.1, 0.6)	0.2 (0.1, 0.4)
Asian	0.6 (0.3, 1.2)	1.1 (0.6, 2.5)
Median household income	47,790.5 (40,718.0, 55,627.3)	50,249.5 (42,840.3, 60,139.3)
Unemployed %^b^	6.3 (4.4, 8.7)	6.8 (5.2, 8.8)
Families in poverty %^c^	10.7 (7.6, 14.5)	11.1 (7.9, 14.3)
Population density^d^	0.05 (0.02, 0.17)	0.14 (0.06, 0.36)
**Policies**	**Counts and Proportion**	**Counts and Proportion**
PDMP must‐query		
2013	175 (5.6)	41 (3.1)
2020	2,016 (64.3)	1,055 (78.7)
PMC laws with penalties		
2013	187 (6.0)	95 (7.1)
2020	761 (24.3)	408 (30.4)
Overdose Good Samaritan laws		
2013	357 (11.4)	176 (13.1)
2020	2,733 (87.2)	1,188 (88.7)
Naloxone access laws		
2013	307 (9.8)	178 (13.3)
2020	3,134 (100.0)	1,340 (100.0)
Medicaid expansion		
2013	0 (0.0)	0 (0.0)
2020	1,710 (54.6)	697 (52.0)

Abbreviations: IQ, interquartile; PDMP, prescription drug monitoring programs; PMC, pain management clinic.

^a^ Counties during the study period that have at least 500 Black, Non‐Hispanic residents and 500 Hispanic residents.

^b^ Percentage unemployed in civic labor force.

^c^ Percentage families living in poverty within the last 12 months.

^d^ Defined as 1,000 persons per square mile.

### All Overdoses

Adoption of must‐query law was associated with increases in county‐level relative rates of total overdose deaths per 100,000 (adjusted RR = 1.11, 95% CrI: 1.08‐1.14). Similar increases were seen for both the White and Black opioid overdose death rate (RR = 1.10, 95% CrI: 1.08‐1.13 and RR = 1.11, 95% CrI: 1.06‐1.18, respectively). There were larger increases for Hispanic individuals (RR = 1.24, 95% CrI: 1.16‐1.33) (Figure 3).

**Figure 3:**
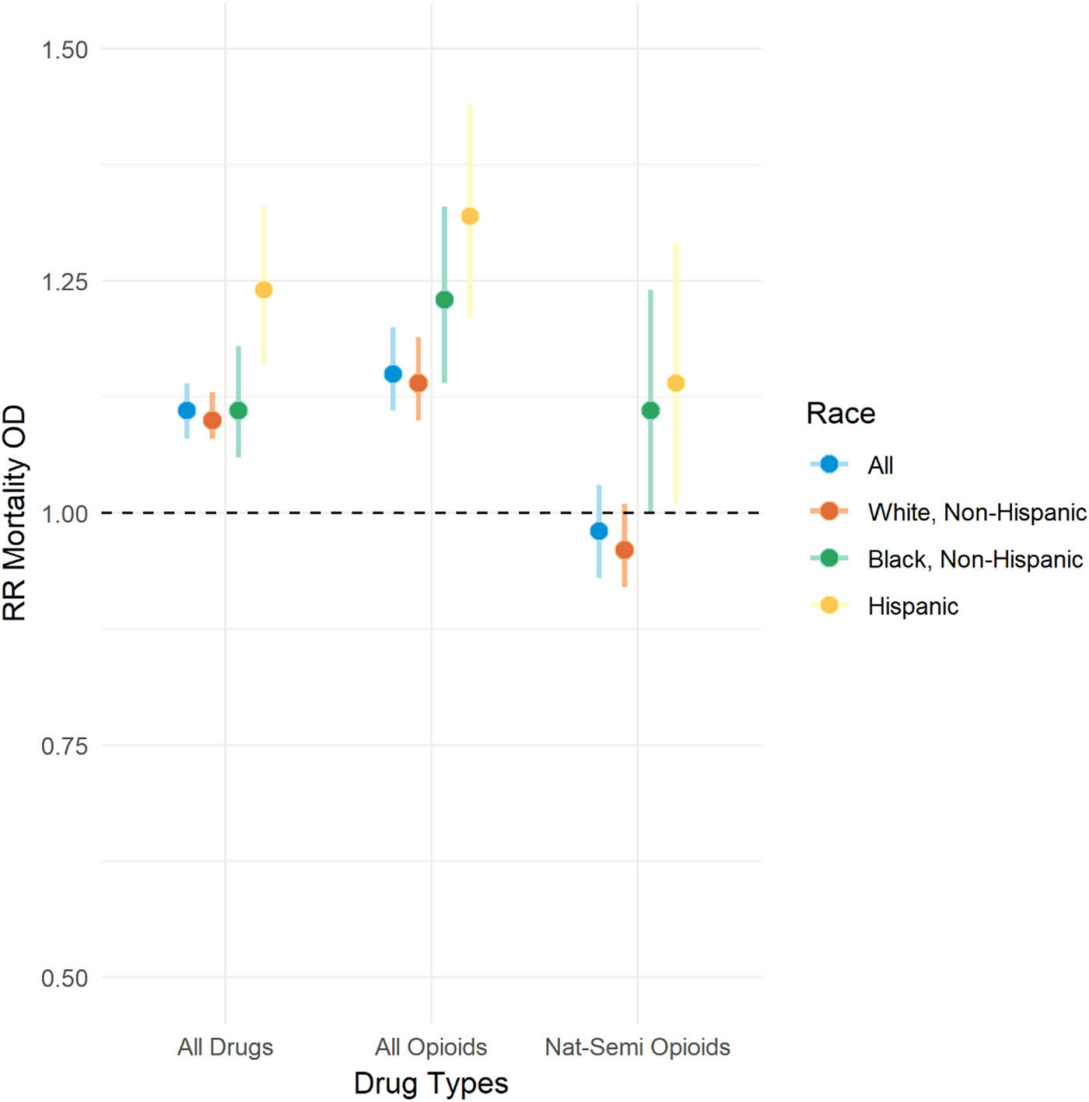
RR and 95% CrIs for Must‐Query PDMPs During the Study Period in the United States Stratified by Drug Overdoses by Race and Ethnicity, 2013 to 2020. CrI, credible interval; OD, overdose; PDMP, prescription drug monitoring program; RR, relative rate. [Colour figure can be viewed at wileyonlinelibrary.com]

### All Opioids

Adoption of must‐query law was associated with increases in county‐level relative rates of total opioid overdose deaths (adjusted RR = 1.15, 95% CrI: 1.11‐1.20). Similar increases were seen for the White opioid overdose death rate (RR = 1.14, 95% CrI: 1.10‐1.19). Larger increases were apparent for Black and Hispanic individuals (RR = 1.23, 95% CrI: 1.14‐1.33 and RR = 1.32, 95% CrI: 1.21‐1.44, respectively; Figure 3).

### Natural and Semisynthetic Opioids (e.g., Oxycodone, Hydrocodone, Oxymorphone, Hydromorphone)

Results for the impact of must‐query laws on the relative rate of natural and semisynthetic opioid overdoses were in the protective direction, although not statistically significant for overall and non‐Hispanic White individuals (RR = 0.98, 95% CrI: 0.93‐1.03 and RR = 0.96, 95% CrI: 0.92‐1.01, respectively). However, must‐query mandates were associated with increased relative risk of natural and semisynthetic opioid overdoses for Black individuals (RR = 1.11, 95% CrI: 1.00‐1.24) and Hispanic individuals (RR = 1.14, 95% CrI: 1.01‐1.29; Table  2 and Figure 3).

**Table 2:** Spatiotemporal Primary Outcome Models by Overdose Drug Type and Race and Ethnicity in the United States, 2013 to 2020

All Drug Overdoses	RRs and CrIs			
Policy	Total	White, Non‐Hispanic	Black, Non‐Hispanic	Hispanic
PDMP must‐query	1.11 (1.08‐1.14)	1.10 (1.08‐1.13)	1.11 (1.06‐1.18)	1.24 (1.16‐1.33)
PMC laws with penalties	0.85 (0.80‐0.90)	0.85 (0.80‐0.91)	0.86 (0.75‐1.00)	0.72 (0.59‐0.89)
Overdose Good Samaritan laws	0.96 (0.94‐0.99)	0.96 (0.94‐0.99)	0.97 (0.91‐1.03)	1.07 (0.99‐1.17)
Naloxone access laws	1.05 (1.02‐1.08)	1.06 (1.03‐1.10)	1.08 (1.00‐1.15)	1.15 (1.06‐1.25)
Medicaid expansion	1.09 (1.05‐1.12)	1.08 (1.05‐1.12)	1.01 (0.94‐1.08)	0.91 (0.82‐1.00)
**All Opioid Overdoses**	**RRs and CrIs**			
**Policy**	**Total**	**White, Non‐Hispanic**	**Black, Non‐Hispanic**	**Hispanic**
PDMP must‐query	1.15 (1.11‐1.20)	1.14 (1.10‐1.19)	1.23 (1.14‐1.33)	1.32 (1.21‐1.44)
PMC laws with penalties	0.89 (0.81‐0.87)	0.90 (0.81‐0.99)	0.85 (0.68‐1.07)	0.58 (0.44‐0.76)
Overdose Good Samaritan laws	0.97 (0.93‐1.01)	0.97 (0.93‐1.01)	0.90 (0.82‐0.99)	1.03 (0.93‐1.14)
Naloxone access laws	1.16 (1.11‐1.22)	1.17 (1.12‐1.23)	1.29 (1.16‐1.43)	1.31 (1.17‐1.47)
Medicaid expansion	1.13 (1.08‐1.19)	1.13 (1.07‐1.18)	1.05 (0.95‐1.17)	0.88 (0.77‐1.00)
**Natural and Semisynthetic Opioid Overdoses** ^a^	**RRs and CrIs**			
**Policy**	**Total**	**White, Non‐Hispanic**	**Black, Non‐Hispanic**	**Hispanic**
PDMP must‐query	0.98 (0.93‐1.03)	0.96 (0.92‐1.01)	1.11 (1.00‐1.24)	1.14 (1.01‐1.29)
PMC laws with penalties	1.10 (0.97‐1.23)	1.08 (0.96‐1.22)	1.06 (0.78‐1.45)	0.93 (0.61‐1.42)
Overdose Good Samaritan laws	0.91 (0.86‐0.96)	0.92 (0.87‐0.97)	0.90 (0.79‐1.03)	0.88 (0.75‐1.04)
Naloxone access laws	1.19 (1.12‐1.27)	1.18 (1.11‐1.26)	1.36 (1.19‐1.56)	1.33 (1.14‐1.56)
Medicaid expansion	1.15 (1.09‐1.22)	1.14 (1.07‐1.21)	1.16 (1.01‐1.33)	1.03 (0.89‐1.20)

Abbreviations: CrI, credible interval; PDMP, prescription drug monitoring programs; PMC, pain management clinic; RR, relative rate.

^a^ Includes opioids such as oxycodone, hydrocodone, oxymorphone, and hydromorphone.

**Table 3:** County Characteristics Stratified by Counties With At Least 1,000 Persons of Specified Race and Ethnicity (Median and IQ Range), United States, 2013 to 2020

Characteristic	Total^a^ (*n* = 3,095)	White, Non‐Hispanic^a^ (*n* = 2,809)	Black, Non‐Hispanic^a^ (*n* = 1,396)	Hispanic^a^ (*n* = 1,492)
	Median (IQ Range)	Median (IQ Range)	Median (IQ Range)	Median (IQ Range)
Age group %, y				
0‐17	22.5 (20.5, 24.4)	22.6 (20.6, 24.3)	22.6 (20.8, 24.4)	23.2 (21.2, 25.1)
18‐24	8.2 (7.3, 9.4)	8.2 (7.4, 9.5)	8.8 (8.0, 10.0)	8.7 (7.9, 10.0)
25‐44	23.3 (21.5, 25.2)	23.5 (21.9, 25.3)	24.6 (23.0, 26.4)	24.5 (22.9, 26.3)
45‐64	27.5 (25.8)	27.5 (25.8, 29.0)	26.9 (25.4, 28.3)	26.6 (24.8, 28.2)
65 and older	17.4 (14.7, 20.2)	17.1 (14.6, 19.7)	15.9 (13.6, 18.1)	15.6 (13.2, 18.0)
Sex %				
Female	50.4 (49.5, 51.1)	50.5 (49.6, 51.1)	50.9 (50.0, 51.6)	50.6 (49.8, 51.2)
Male	49.6 (48.9, 50.5)	49.5 (48.9, 50.4)	49.1 (48.4, 50.0)	49.4 (48.8, 50.2)
Race and ethnicity %				
White, non‐Hispanic	84.3 (65.2, 92.9)	83.6 (65.1, 92.6)	70.6 (56.7, 83.3)	75.2 (59.6, 85.6)
Black, non‐Hispanic	2.2 (0.6, 10.2)	2.7 (0.7, 11.7)	11.9 (5.0, 27.8)	4.3 (1.3, 12.7)
Hispanic	3.9 (2.0, 9.2)	3.9 (2.0, 9.0)	4.9 (2.7, 10.1)	8.1 (4.5, 17.4)
Native American	0.3 (0.1, 0.6)	0.3 (0.1, 0.6)	0.2 (0.1, 0.4)	0.3 (0.1, 0.6)
Asian	0.6 (0.3, 1.2)	0.6 (0.3, 1.3)	1.0 (0.5, 2.3)	1.1 (0.6, 2.3)
Median household income	47,788.5 (40,718.0, 55,605.3)	47,983.0 (40,794.8, 55,993.3)	47,843.0 (40,026.8, 58,366.3)	51,083.0 (43,919.8, 60,095.5)
Unemployed %^b^	6.2 (4.4, 8.5)	6.5 (4.8, 8.8)	7.2 (5.5, 9.4)	6.5 (5.0, 8.6)
Families in poverty %^c^	10.6 (7.6, 14.2)	10.9 (7.8, 14.6)	12.0 (8.6, 16.0)	10.6 (7.6, 13.7)
Population density^4^	0.05 (0.02, 0.12)	0.05 (0.02, 0.1)	0.1 (0.05, 0.3)	0.1 (0.04, 0.3)
**Policies**	**Count and Proportion**	**Count and Proportion**	**Count and Proportion**	**Count and Proportion**
PDMP must‐query				
2013	165 (5.3)	292 (10.4)	97 (7.0)	94 (6.3)
2020	2,010 (64.9)	1,899 (67.6)	1,171 (83.9)	1,003 (67.2)
PMC with penalties				
2013	187 (6.0)	183 (6.5)	99 (7.1)	76 (5.1)
2020	754 (24.4)	693 (24.7)	454 (32.5)	415 (27.8)
Overdose Good Samaritan laws				
2013	351 (11.3)	591 (21.0)	379 (27.2)	449 (30.1)
2020	2,705 (87.4)	2,509 (89.3)	1,273 (91.2)	1,254 (84.0)
Naloxone access laws				
2013	306 (9.9)	602 (21.4)	305 (21.9)	395 (26.5)
2020	3,095 (100.0)	2,809 (100.0)	1,396 (100.0)	1,492 (100.0)
Medicaid expansion				
2013	0 (0.0)	0 (0.0)	0 (0.0)	0 (0.0)
2020	1,695 (54.8)	1,522 (54.2)	678 (48.6)	767 (51.4)

Abbreviations: IQ interquartile; PDMP prescription drug monitoring programs; PMC, pain management clinic.

^a^ Counties during the study period that have at least 1,000 residents specified within the column.

^b^ Percentage unemployed in civic labor force.

^c^ Percentage families living in poverty within the last 12 months.

^d^Defined as 1,000 persons per square mile.

## Sensitivity Results

Results were robust to different specifications of counties included in the race‐stratified analyses, (Table 3), except for the impact of PDMPs on natural/semisynthetic opioids for White individuals. In the sensitivity analysis, when considering a larger sample of counties, there was a protective effect of PDMPs.

## Discussion

This study examined the association between the implementation of “must‐query” PDMPs and county‐level overdose rates in the United States from 2013 to 2020. PDMPs are a supply‐side form of opioid regulation aimed at reducing adverse outcomes related to prescription opioids (natural and semisynthetic opioid overdoses). From 2013 to 2020, when considering natural and semisynthetic opioid overdoses, there was no overall impact of PDMP must‐query mandates overall on overdose deaths. However, when considering overdose deaths attributable other causes, such as all overdoses and all opioid overdoses, adoption of must‐query law was associated with increases in county‐level relative rates of both. These findings indicate the unintended consequences of PDMPs.^38^, ^39^, ^40^, ^41^ Greater restrictions on the prescribing and distribution of prescription opioids may have inadvertently contributed to a shift in opioid‐use patterns, leading some individuals with prescription opioid dependence to transition to heroin or illicitly manufactured synthetic opioids such as fentanyl.

However, the adoption of must‐query PDMPs had differential impacts on natural and semisynthetic overdoses by race and ethnicity. There was no appreciable impact among White individuals in the main study sample. Sensitivity analysis showed that there was a protective impact for White individuals. In contrast, Black and Hispanic individuals experienced an increase in overdose rates for the same drug category following must‐query PDMP adoption. In addition, the magnitude of the increase in overall overdose deaths and opioid overdose deaths associated with must‐query PDMP adoption was greater among Black and Hispanic individuals. Hispanic and Black individuals experienced the largest relative increases in overdose rates. These findings suggest that although PDMPs may reduce the negative consequences of prescription opioids (natural and semisynthetic opioids) among some populations, they may not have equal impacts across all racial and ethnic groups.

The finding that must‐query PDMP adoption was associated with increased natural and semisynthetic opioid overdose deaths among Black and Hispanic populations despite no appreciable change among White individuals warrants careful consideration. Our results underscore the importance of considering racial and ethnic differences when evaluating the effectiveness of PDMPs. The observed increases in overdose rates among Black and Hispanic individuals suggest that PDMPs may not uniformly benefit all populations. These disparities may reflect broader systemic inequities in health care access, pain management, and substance use treatment.^42^, ^43^, ^44^, ^45^ For instance, Black and Hispanic patients were less likely than White patients to be prescribed these medications for legitimate pain needs.^46^ When PDMP mandates were introduced, prescribers might have disproportionately reduced or discontinued prescribing for these patients, potentially pushing some toward unregulated drug supplies in which potency and contamination risks are higher. Differential surveillance and enforcement in communities of color may have amplified the impact of PDMP mandates, with greater scrutiny leading to abrupt disruptions in opioid access and fewer opportunities for safe tapering or linkage to treatment. Additionally, people of color are more likely to encounter barriers to accessing medication‐assisted treatment (MAT), leaving Black and Hispanic individuals more vulnerable to the consequences of prescription opioid supply reductions.^43^, ^45^ Without affordable and accessible health care options, individuals may resort to self‐managing pain or substance use disorders, increasing the likelihood of overdose. Additionally, stigma surrounding substance use disorders remains a pervasive issue, particularly for individuals in marginalized racial and ethnic groups.^47^ Research has shown that Black and Hispanic individuals are more likely to be perceived as engaging in criminal behavior rather than as needing medical treatment when seeking care for substance use.^48^, ^49^ This stigma can deter individuals from accessing harm‐reduction services, such as MAT or naloxone distribution programs. When must‐query PDMP mandates reduce access to prescription opioids without simultaneously addressing these structural inequities, individuals in these circumstances are left with few safe alternatives, increasing their vulnerability to overdose. As the individuals who are most burdened by the overdose crisis shift, there is need to ensure equitable access to treatment to mitigate these harms.

This study has several limitations that should be considered. First, we could not include all counties in our analysis, given the unequal distribution of racial and ethnic groups across counties in the United States. Counties without heterogeneity in their racial and ethnic makeup may be systematically different than racially heterogeneous counties. However, sensitivity analysis including all possible counties resulted in similar findings. Second, we were unable to examine the association among American Indian/Alaskan Native individuals, a group experiencing some of the highest rates of fatal overdose, because of data sparseness. Third, our study is not able to make causal claims on the association between “must‐query” PDMP and fatal overdose; although we controlled for key policy covariates, unmeasured confounding factors such as local economic conditions and availability of illicit drugs could influence the results. Fourth, this study focused on the impact of PDMPs on fatal opioid overdoses. Future studies should consider the impact of PDMPs on the spectrum of opioid‐related harm, including but not limited to nonfatal overdoses and potential effects of overdose from other opioid types. Fifth, even with adjustment for other contemporaneous policies, the close timing of multiple laws limit the ability to fully isolate the independent effect of must‐query PDMP laws. Finally, variation exists across states and years in the completeness of reporting specific drugs on death certificates, which could lead to misclassification of drug‐involved overdose deaths and potentially bias our estimates.^50^

Future research should explore the mechanisms driving racial and ethnic disparities in PDMP effectiveness, especially given the increasing rates of overdose deaths among Black and Hispanic individuals caused by natural and semisynthetic opioids. Studies incorporating individual‐level data and qualitative analyses could provide deeper insights into the lived experiences of patients and prescribers under must‐query mandates. Additionally, evaluating the interplay between PDMPs and other harm reduction strategies, such as naloxone distribution and treatment for substance use disorder, could help identify more holistic solutions to the overdose crisis.

Our results indicate that must‐query PDMP provisions may not reduce opioid overdose mortality equitably and, in some cases, may be associated with greater increases among Black and Hispanic populations. These findings underscore the need to critically assess whether PDMPs, as currently implemented, are effective in reducing overdose deaths across all racial and ethnic groups and whether additional or alternative interventions are necessary to prevent the exacerbation of existing disparities. Such evidence could inform policy decisions on the modification or replacement of must‐query PDMP provisions in the context of evolving overdose trends. Additionally, addressing systemic inequities in health care access and substance use treatment is essential to mitigating the unintended consequences of PDMP implementation and reducing the burden of drug overdose deaths overall.

## References

[1] Spencer MR , Garnett MF , Miniño AM . Drug overdose deaths in the United States, 2002‐2022. NCHS Data Briefs. 2024(491). 10.15620/cdc:135849 PMC12278041 40623710

[2] Drug overdose deaths: facts and figures . National Institute on Drug Abuse. August 21, 2024. Accessed December 31, 2024. https://nida.nih.gov/research‐topics/trends‐statistics/overdose‐death‐rates

[3] Overdose prevention: prescription drug monitoring programs (PDMPs) . Centers for Disease Control and Prevention. May 21, 2024. Accessed January 6, 2025. https://www.cdc.gov/overdose‐prevention/php/interventions/prescription‐drug‐monitoring‐programs.html

[4] D'Souza RS , Lang M , Eldrige JS . Prescription drug monitoring program. In: StatPearls. Treasure Island: StatPearls Publishing; 2024. 30335332

[5] Delcher C , Wagenaar AC , Goldberger BA , Cook RL , Maldonado‐Molina MM . Abrupt decline in oxycodone‐caused mortality after implementation of Florida's prescription drug monitoring program. Drug Alcohol Depend. 2015;150:63‐68. 10.1016/j.drugalcdep.2015.02.010 25746236

[6] Kilby AE . Opioids for the Masses: Welfare Tradeoffs in the Regulation of Narcotic Pain Medications . Dissertation. Massachusetts Institute of Technology; 2016. Accessed March 6, 2025. https://angelakilby.com/pdfs/AKilbyWelfare_2022‐01.pdf

[7] Pardo B . Do more robust prescription drug monitoring programs reduce prescription opioid overdose? Addiction. 2017;112(10):1773‐1783. 10.1111/add.13741 28009931

[8] Li G , Brady JE , Lang BH , Giglio J , DiMaggio C . Prescription drug monitoring and drug overdose mortality. Inj Epidemiol. 2014;1(1):9. 10.1186/2197-1714-1-9 27747666 PMC5005551

[9] Nam YH , Shea DG , Shi Y , Moran JR . State prescription drug monitoring programs and fatal drug overdoses. Am J Manag Care. 2017;23(5):297‐303. 28738683

[10] Paulozzi LJ , Kilbourne EM , Desai HA . Prescription drug monitoring programs and death rates from drug overdose. Pain Med. 2011;12(5):747‐754. 10.1111/j.1526-4637.2011.01062.x 21332934

[11] Lee B , Zhao W , Yang KC , Ahn YY , Perry BL . Systematic evaluation of state policy interventions targeting the US opioid epidemic, 2007‐2018. JAMA Netw Open. 2021;4(2):e2036687. 10.1001/jamanetworkopen.2020.36687 33576816 PMC7881356

[12] Kreiner P , Nikitin R , Shields TP . Bureau of Justice Assistance Prescription Drug Monitoring Program Performance Measures Report: January 2009 Through June 2012 . US Department of Justice, Bureau of Justice Assistance; 2014.

[13] Mandating PDMP Participation by Medical Providers: Current Status and Experience in Selected States. Prescription Drug Monitoring Center of Excellence at Brandeis; 2014.

[14] Meinhofer A . Prescription drug monitoring programs: the role of asymmetric information on drug availability and abuse. Am J Health Econ. 2018;4(4):504‐526. 10.1162/ajhe_a_00101

[15] Birk E , Waddell GR . The mitigating role of prescription drug monitoring programs in the abuse of prescription drugs. IZA Discussion Paper No. 10990. Institute for the Study of Labor (IZA); 2017. 10.2139/ssrn.3037875

[16] Cerdá M , Wheeler‐Martin K , Bruzelius E , et al. The role of prescription opioid and cannabis supply policies on opioid overdose deaths. Am J Epidemiol. 2025;194(3):791‐801. 10.1093/aje/kwae210 39030721 PMC12168759

[17] Pauly NJ , Slavova S , Delcher C , Freeman PR , Talbert J . Features of prescription drug monitoring programs associated with reduced rates of prescription opioid‐related poisonings. Drug Alcohol Depend. 2018;184:26‐32. 10.1016/j.drugalcdep.2017.12.002 29402676 PMC5854200

[18] Phillips E , Gazmararian J . Implications of prescription drug monitoring and medical cannabis legislation on opioid overdose mortality. J Opioid Manag. 2017;13(4):229‐239. 10.5055/jom.2017.0391 28953315

[19] Brown R , Riley MR , Ulrich L , et al. Impact of New York prescription drug monitoring program, I‐STOP, on statewide overdose morbidity. Drug Alcohol Depend. 2017;178:348‐354. 10.1016/j.drugalcdep.2017.05.023 28692945

[20] Patrick SW , Fry CE , Jones TF , Buntin MB . Implementation of prescription drug monitoring programs associated with reductions in opioid‐related death rates. Health Aff (Millwood). 2016;35(7):1324‐1332. 10.1377/hlthaff.2015.1496 27335101 PMC5155336

[21] Townsend TN , Bohnert ASB , Lagisetty P , Haffajee RL . Did prescribing laws disproportionately affect opioid dispensing to Black patients? Health Serv Res. 2022;57(3):482‐496. 10.1111/1475-6773.13968 35243639 PMC9108058

[22] Friedman JR , Hansen H . Evaluation of increases in drug overdose mortality rates in the US by race and ethnicity before and during the COVID‐19 pandemic. JAMA Psychiatry. 2022;79(4):379‐381. 10.1001/jamapsychiatry.2022.0004 35234815 PMC8892360

[23] Restricted‐use vital statistics data . National Center for Health Statistics. Centers for Disease Control and Prevention. August 24, 2023. Accessed March 6, 2025. https://www.cdc.gov/nchs/nvss/nvss‐restricted‐data.htm

[24] Injury Surveillance Workgroup (ISW) reports . Safe States Alliance website. Accessed September 16, 2024. https://www.safestates.org/page/ISWReports

[25] Horwitz JR , Davis C , McClelland L , Fordon R , Meara E . The importance of data source in prescription drug monitoring program research. Health Serv Res. 2021;56(2):268‐274. 10.1111/1475-6773.13548 32880934 PMC7968938

[26] McClellan C , Lambdin BH , Ali MM , et al. Opioid‐overdose laws association with opioid use and overdose mortality. Addict Behav. 2018;86:90‐95. 10.1016/j.addbeh.2018.03.014 29610001

[27] Cerdá M , Wheeler‐Martin K , Bruzelius E , et al. Spatiotemporal analysis of the association between pain management clinic laws and opioid prescribing and overdose deaths. Am J Epidemiol. 2021;190(12):2592‐2603. 10.1093/aje/kwab192 34216209 PMC8796812

[28] Prescription Drug Abuse Policy System ‐ PDAPS . PDAPS website. Accessed August 14, 2024. https://pdaps.org/

[29] Status of state Medicaid expansion decisions. Kaiser Family Foundation . February 12, 2025. Accessed March 6, 2025. https://www.kff.org/status‐of‐state‐medicaid‐expansion‐decisions/

[30] American community survey 5‐year data (2009‐2023) . US Census Bureau website. December 12, 2024. Accessed March 6, 2025. https://www.census.gov/data/developers/data‐sets/acs‐5year.html

[31] Bernardinelli L , Clayton D , Pascutto C , Montomoli C , Ghislandi M , Songini M . Bayesian analysis of space‐time variation in disease risk. Stat Med. 1995;14(21‐22):2433‐2443. https://pubmed.ncbi.nlm.nih.gov/8711279/ 8711279 10.1002/sim.4780142112

[32] Blangiardo M , Cameletti M , Baio G , Rue H . Spatial and spatio‐temporal models with R‐INLA. Spat Spatiotemporal Epidemiol. 2013;4:33‐49. 10.1016/j.sste.2012.12.001 23481252

[33] Lord D , Washington SP , Ivan JN . Poisson, Poisson‐gamma and zero‐inflated regression models of motor vehicle crashes: balancing statistical fit and theory. Accid Anal Prev. 2005;37(1):35‐46. 10.1016/j.aap.2004.02.004 15607273

[34] Banerjee S , Carlin BP , Gelfand AE . Hierarchical Modeling and Analysis for Spatial Data. CRC Press; 2003.

[35] Bakka H , Rue H , Fuglstad GA , et al. Spatial modeling with R‐INLA: a review. WIREs Computational Statistics. 2018;10(6):e1443. 10.1002/wics.1443

[36] R: A language and environment for statistical computing . Version 4.1.1. R; 2010.

[37] DiMaggio C . Spatial epidemiology notes. Appl Vignettes R. 2012;20(2):242‐244.

[38] Cerdá M , Ponicki WR , Smith N , et al. Measuring relationships between proactive reporting state‐level prescription drug monitoring programs and county‐level fatal prescription opioid overdoses. Epidemiology. 2020;31(1):32‐42. 10.1097/EDE.0000000000001123 31596794 PMC7027962

[39] Martins SS , Ponicki W , Smith N , et al. Prescription drug monitoring programs operational characteristics and fatal heroin poisoning. Int J Drug Policy. 2019;74:174‐180. 10.1016/j.drugpo.2019.10.001 31627159 PMC6897357

[40] Martins SS , Bruzelius E , Stingone JA , et al. Prescription opioid laws and opioid dispensing in US counties: identifying salient law provisions with machine learning. Epidemiology. 2021;32(6):868. 10.1097/EDE.0000000000001404 34310445 PMC8556655

[41] Yuanhong Lai A , Smith KC , Vernick JS , Davis CS , Caleb Alexander G , Rutkow L . Perceived unintended consequences of prescription drug monitoring programs. Subst Use Misuse. 2019;54(2):345‐349. 30463465 10.1080/10826084.2018.1491052

[42] Jegede O , Bellamy C , Jordan A . Systemic racism as a determinant of health inequities for people with substance use disorder. JAMA Psychiatry. 2024;81(3):225‐226. 38231489 10.1001/jamapsychiatry.2023.4958

[43] Stahler GJ , Mennis J . Treatment outcome disparities for opioid users: are there racial and ethnic differences in treatment completion across large US metropolitan areas? Drug Alcohol Depend. 2018;190:170‐178. 30041092 10.1016/j.drugalcdep.2018.06.006

[44] Knoebel RW , Starck JV , Miller P . Treatment disparities among the Black population and their influence on the equitable management of chronic pain. Health Equity. 2021;5(1):596‐605. 34909526 10.1089/heq.2020.0062 PMC8665804

[45] Anderson A , Walker B , Shao Y , LaVeist TA , Callison K . Racial and ethnic disparities in medication‐assisted treatment: evidence from Louisiana Medicaid during the COVID‐19 pandemic. J Gen Int Med. 2023;38(1):266‐268. 10.1007/s11606-022-07893-8 PMC9640815 36344642

[46] Cintron A , Morrison RS . Pain and ethnicity in the United States: a systematic review. J Palliat Med. 2006;9(6):1454‐1473. 10.1089/jpm.2006.9.1454 17187552

[47] Walters SM , Kerr J , Cano M , Earnshaw V , Link B . Intersectional stigma as a fundamental cause of health disparities: A case study of how drug use stigma intersecting with racism and xenophobia creates health inequities for Black and Hispanic persons who use drugs over time. Stigma Health. 2023;8(3):325‐343. 37744082 10.1037/sah0000426 PMC10516303

[48] Kuettel BT . Perceptions of substance use disorder and associated sanctions: a factorial vignette experiment. Crim Justice Rev. 2023;48(2):203‐231.

[49] Netherland J , Hansen HB . The war on drugs that wasn't: wasted whiteness,“dirty doctors,” and race in media coverage of prescription opioid misuse. Cult Med Psychiatry. 2016;40:664‐686. 27272904 10.1007/s11013-016-9496-5 PMC5121004

[50] Jones CM , Warner M , Hedegaard H , Compton W . Data quality considerations when using county‐level opioid overdose death rates to inform policy and practice. Drug Alcohol Depend. 2019;204:107549. 10.1016/j.drugalcdep.2019.107549 31518886 PMC9190240

